# A Comparative Study on “*Mai*” and “Blood Vessels” in Early Chinese and Western Medicine: Based on* Hippocratic Corpus* and* Cauterization Canon of the Eleven Vessels of the Foot and Forearm*

**DOI:** 10.1155/2019/7826234

**Published:** 2019-06-09

**Authors:** Chang Huang, Jiankang Liang, Qicheng Zhang, Tao Lu

**Affiliations:** Beijing University of Chinese Medicine, Beijing 100029, China

## Abstract

This study compared the theory describing the “four pairs of blood vessels” in the* Hippocratic Corpus* with the description of vessels (*Mai*, 脉) in the* Cauterization Canon of the Eleven Vessels of the Foot and Forearm*. The two theories are comparable because of the time period in which they were written, the similarities between the descriptions of the* Mai* and blood vessels, and the treatment methods for symptoms corresponding to their dysfunctions. We discovered that the* Mai* theory and the blood vessel theory in the* Hippocratic Corpus* were conceived with similar motivations. They had a lot of coinciding information with regard to the route of flow, but they proposed opposite cyclic directions. Interestingly, neither of them had established that a definitive relationship exists between the vessels and the heart, but other internal organs, such as the liver, were considered to have connections with the* Mai *and blood vessels in the two literatures. Furthermore, there were similarities among the descriptions of symptoms, and the ancient Western treatments for these symptoms were largely the same as those recorded in the ancient Chinese medical literature, especially the treatment for backache. The comparisons put forward in this study not only reflect the consistencies between the understanding of the human body and the way diseases were treated in Chinese and Western medicine in the early days, but also demonstrate that the two types of medicine had finally embarked on different developmental paths because of the differences in the philosophies and cultural backgrounds in their respective regions.

## 1. Introduction

Hippocrates (around 460–377 BC) was a physician from the Periclean age of ancient Greece. He was the founder of Western medicine who was revered by the West as the “father of medicine” [1]. The* Hippocratic Corpus*, named after him, represented the main achievements of ancient Western medicine. The publication of this corpus symbolized that medicine became an independent discipline in ancient Greece. The treatise named* On Human Nature*, the most famous Hippocratic work, described the routes of the four pairs of major blood vessels and the use of bloodletting therapy to treat symptoms corresponding to their dysfunctions [2]. These descriptions easily reminded people of the descriptions of vessels and bloodletting therapy in the practice of traditional Chinese medicine that had been widely recognized and used in China during the Warring States Period (476–221 BC). In ancient times, owing to the separation between the two geographic regions, there was barely any communication between the Eastern and Western medical worlds. Could there be a wonderful consensus on the understanding of the body and treatment of diseases between them? How did these similarities and differences influence the development of the field of medicine in these two regions? These are interesting research topics.

In previous studies, the* Hippocratic Corpus* had often been compared to China's* The Yellow Emperor's Classic of Medicine *(*Huangdi Neijing*, 黄帝内经), which was regarded as a comparative basis for research on the connections and differences between Chinese and Western medicine because the two books were highly comparable in terms of the significant roles they played in the medical histories of their respective regions [3, 4].* The Yellow Emperor's Classic of Medicine *was considered as the greatest achievement of ancient Chinese medicine, marking the formal establishment of the theoretical system of traditional Chinese medicine [5]. Since the early 20th century, a large number of documents have been unearthed throughout China, including many medical books. According to the researches that unearthed the situations, contents, characters, writings, and other characteristics associated with these medical books, it was known that their publication dates were generally earlier than that of* The Yellow Emperor's Classic of Medicine*, and that some of them were regarded as master copies of this classic; this could help us learn more about the views and medical practices of early physicians in ancient China.

In 1973, medical classics on silk scrolls were unearthed from tomb No. 3 in* Mawangdui *(马王堆). Among these unearthed literatures, the term* Mai* appeared for the first time in the history of traditional Chinese medicine in the* Cauterization Canon of the Eleven Vessels of the Foot and Forearm *(*Zubi Shiyi Maijiujing*, 足臂十一脉灸经). In addition,* Mai *was also talked about in many other unearthed documents, including the* Cauterization Canon of the Eleven Yin and Yang Vessels *(*Yinyang Shiyi Maijiujing*, 阴阳十一脉灸经) and* the Book of the Vessels *(*Maishu*, 脉书), which was a medical book discovered in the* Zhangjiashan *(张家山)* Han* tomb,* Hubei* Province, China, and so on [6]. Many scholars agreed that the* Mai* system was an embryonic form of the* meridian* system (*Jingmai*, 经脉), and the connections between the concepts of* Mai* and the* five viscera and six entrails *(*Wuzang Liufu*, 五脏六腑) laid the foundation for the development of the concept of* Mai *toward the concept of the* meridians* in* The Yellow Emperor's Classic of Medicine* [7]. As the early descriptions of* Mai* contained in the history of traditional Chinese medicine are very close to the contents describing blood vessels in the* Hippocratic Corpus*, this article attempts to discuss the relationship between ancient Chinese and Western medicine by comparing these contents, and the primary comparable contents between these two literatures were compiled in* Supplementary Tables*  *1* and* Supplementary Table*  *2*, while taking into consideration* The Yellow Emperor's Classic of Medicine* as well.

## 2. The Motives behind the* Mai* and Blood Vessel Theories

*Mai* is a very important concept in traditional Chinese medicine. It was found to be a frequently used concept in several unearthed documents, such as the* Cauterization Canon of the Eleven Vessels of the Foot and Forearm*,* Cauterization Canon of the Eleven Yin and Yang Vessels,* and* Book of the Vessels*. One of the written forms of* Mai* is “
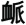
” in the* Cauterization Canon of the Eleven Vessels of the Foot and Forearm*. This Chinese character consists of “血” and “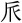.” “血” means “blood,” and “
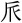
” refers to branches. The explanation in the book,* Explaining Graphs and Analyzing Characters *(*Shuowen Jiezi, *说文解字), which is the oldest authoritative dictionary in China that was written in the* Eastern Han* dynasty (25–220 AD), describes a* Mai* as a branch of blood flow [8]. Thus, the term* Mai *in unearthed literatures refers to blood vessels according to the principle of Chinese word-formation. Moreover, corresponding contents in the* Records of the Grand Historian *(*Shiji, *史记) [9] also supported this statement. In those days, both Chinese and Western medicine agreed that the heart pumps blood through the blood vessels of the circulatory system. However, apart from outlining that the* forearm Greater Yin vessel* terminates at heart, no descriptions were provided about the relationship between the heart and the other ten* Mai*. The most plausible explanation for this was that the eleven* Mai *actually represent the strong affiliations that exist between the extremities and proximal parts of the body [10], rather than the vessels in the circulatory system. We could diagnose and treat diseases affecting proximal parts via the extremities; this idea is in accordance with clinically practiced acupuncture nowadays as well.* Mai*, translated as vessels, is just a borrowed name to represent this affiliation since the vessels are the only anatomical structures that run through the whole body and that can be observed on the body surface. Chinese ancients generalized the concept of the eleven* Mai* in the unearthed literatures, and the number eleven is more likely to cater to the earlier numerological model of the* six factors in heaven and five elements in earth *(*Tianliu Diwu*, 天六地五) [7]. Coincidentally, the blood vessel theory, in a number of its components, is also associated with numerology. The treatise from which the concept of the four pairs of blood vessels comes from was believed to be written by Polybus, the disciple, and son-in-law of Hippocrates [11]. However, many medical theorists harbored negative opinions of Polybus' vessel model, such as Alcmaeon, who suggested that the blood vessels originated from the head and brain [12]. Galen, the most influential medical guru in ancient Rome, who was the first to recognize venous and arterial blood, also objected to Polybus' postulation on vessels [13]. Hence, scholars have proposed that the theory of the four pairs of blood vessels was established on account of the numerology associated with the number four. This theory was set up to be consistent with the four principle elements of the universe and the theory of the four body humors [14]. In this respect, we could identify that the motive behind the reasoning that led to the concept of* Mai* is similar to that which led to the blood vessel theory. Notwithstanding, previous studies have not specified that the four pairs of blood vessels represent a relationship between the different parts of the body like the* Mai *do. We could still get some slight hints on how Polybus understood the function of his understanding of vessels from the symptoms and therapies recorded in* On Human Nature*. It indicated that he had perceived a mysterious connection between the different parts of the body.

## 3. The Routes and Trends of Blood Vessels and Mai

The four pairs of blood vessels in the* Hippocratic Corpus *and the* Mai* in the* Cauterization Canon of the Eleven Vessels of the Foot and Forearm* have a lot in common in their circulatory routes but have opposite travelling directions. All of the blood vessels descend from the head to the limbs. On the contrary, all* Mai* travel from the end of the limbs to the chest, abdomen, or head, which is called “centripetal” circulation. From the perspective of the running routes, the similarities and differences between blood vessels and* Mai* are illustrated below.

**Figure 1:**
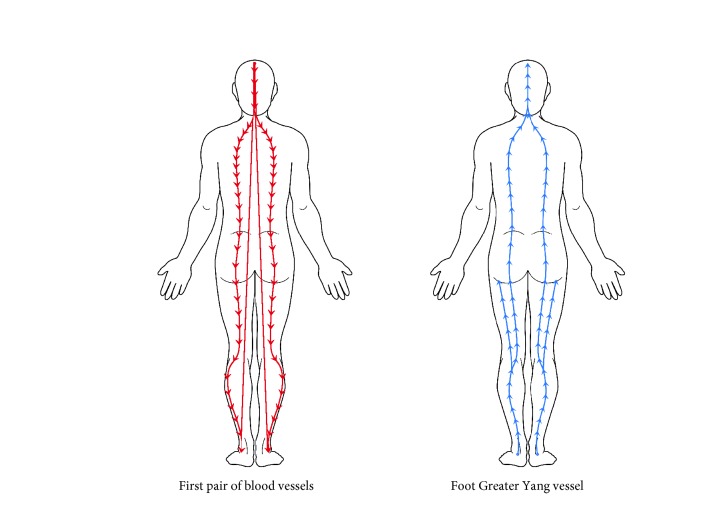
The routes and trends of the first pair of blood vessels and the* foot Great Yang vessel*.

The first pair of blood vessels are a pair of blood vessels that descend from the back of the head to the neck and travel down the backbone on either side until they reach the loins where they branch into the legs, extend to the outer sides of the crus and ankles, and finally enter into the feet. The running route of this pair of blood vessels is very close to that of the* foot Greater Yang vessel* in the* Cauterization Canon of the Eleven Vessels of the Foot and Forearm *(Table 1; Figure 1), and the main difference between them is that the running route of the* foot Greater Yang vessel* is longer and extends from the head to the ears, eyes, and nose.

**Figure 2:**
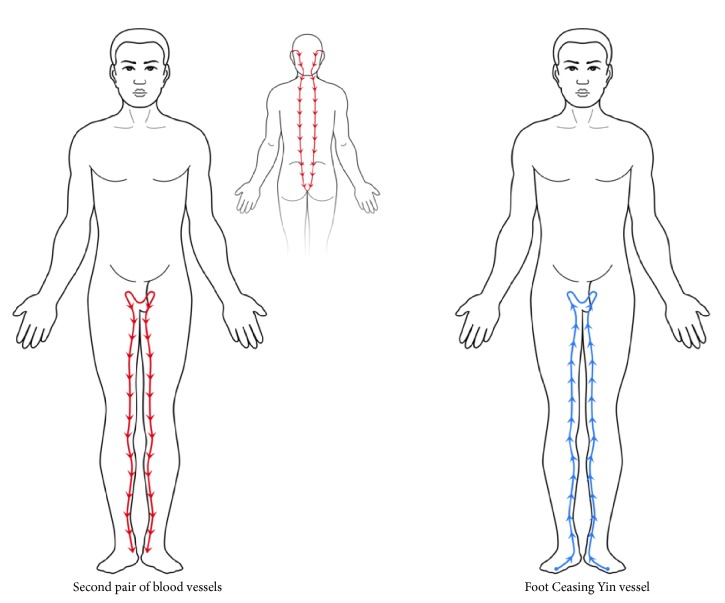
The routes and trends of the second pair of blood vessels and the* foot Ceasing Yin vessel.*

The second pair of blood vessels are a pair of blood vessels that descend from the vicinity of the two ears into the neck, one on each side, and enter into the backbone, travel through its length, and spread into the testicles and thighs along the loins; they then travel down from the medial sides of the thighs and through the cavities behind each knee, then run through the insides of the shins to the insides of the ankles, and finally enter into the feet. The posterior running route of this pair of blood vessels is akin to that of the* foot Ceasing Yin vessel *(Table 2; Figure 2). While the* foot Ceasing Yin vessel *runs from the beginning of the toes to the end of the testicles, the second pair of blood vessels extend further by going through the backbone, neck, and ears.

The third pair of blood vessels are an extraordinary pair of blood vessels that have intersected running routes. When the two vessels descend from the temples to the lungs, the right blood vessel turns to the left, and the left blood vessel turns to the right. Each of the eleven* Mai* are symmetrically located on both sides of the body without crossing paths; therefore, none of the* Mai* have similar running routes to this pair of blood vessels.

**Figure 3:**
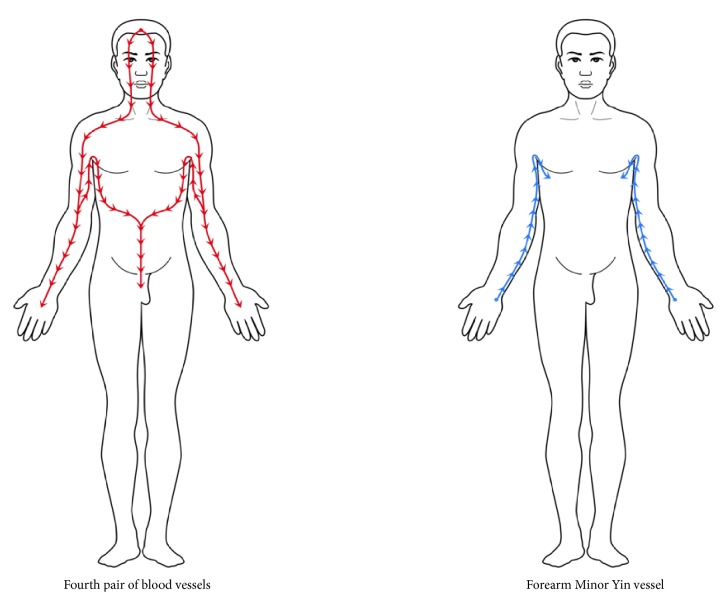
The routes and trends of the fourth pair of blood vessels and the* forearm Minor Yin vessel.*

The last pair of blood vessels are a pair of blood vessels that descend from the front of the head and eyes and travel underneath the collarbones. One vessel runs along the upper part of each upper arm to the elbows and then through the forearms to the palms. The other one stretches from the lower part of each upper arm to the armpits. They then travel above the ribs; one of the vessels reaches the spleen, and the other one reaches the liver. Finally, both vessels on the left and right sides pass over the stomach and terminate at the penis. The paths of the running routes of these blood vessels are very similar to that of the* forearm Minor Yin vessel *(Table 3; Figure 3). It starts from the medial posterior edges of the arm muscles and goes along the cubital fossa posterior. Finally, passing through the armpits, it ends at ribs. From this it is obvious that the last pair of blood vessels have a more complete route, travelling from the head and eyes to the abdomen and penis.

## 4. Relationship with the Organs and Nine Orifices

The descriptions of the blood vessels in the* Hippocratic Corpus *and the* Mai* in the* Cauterization Canon of the Eleven Vessels of the Foot and Forearm* outline their connections with different visceral organs. In ancient Greek, from reading* On the Heart *and other treatises, it could be observed that Hippocrates had realized that the heart was the supplier of blood. He pointed out that the heart has two ventricles and two atriums. The left ventricle supplies blood to the lungs and receives air from the lungs. In* The Yellow Emperor's Classic of Medicine*, it was also believed that the heart dominates blood circulation. However, all four pairs of blood vessels start from the head rather than the heart, while the eleven* Mai* start from the hands and feet. Therefore, it could be inferred that the connotations associated with the blood vessels and* Mai* are not the same as those attributed to vessels nowadays.

The last two pairs of blood vessels that mainly run through the internal body cavity have close connections with the lungs, liver, spleen, and kidneys, but there are fewer internal organs mentioned in the* Cauterization Canon of the Eleven Vessels of the Foot and Forearm*, mainly due to the fact that it had not been established at the time that correspondences exist between the external body parts and internal organs. With the development of the theory of* meridians and collaterals* in traditional Chinese medicine, this concern was gradually resolved.* The Yellow Emperor's Classic of Medicine* describes twelve channels called* meridians,* including their distribution, their physiological functions, and their relationships with the organs. Per this literature, each of the twelve* meridians* belongs to their respective organs, and this revealed that the interior organs and superficial parts of the body interrelate with each other. From this, we could know that both the* Mai* and blood vessels were interconnected with bodily organs and were associated with bodily functions.

There is a concept known as the* nine orifices* (*Jiuqiao*, 九窍) in traditional Chinese medicine. It refers to the holes via which the human body communicates with the outside world. The* nine orifices *include the two eyes, two nostrils, two ears, mouth, anus, and urethra. Eight* Mai* of the* Cauterization Canon of the Eleven Vessels of the Foot and Forearm* end at one or several of these nine orifices, mainly the ones in the head. This illustrates that* Mai *may not be blood vessels circulating in the body but instead are the channels that link up the environment with the internal body.* Qi*, a vital energy, flows through these channels and circulates in the body to maintain health. Through the nine orifices, this type of* Qi* can be influenced by the* Qi* of nature and can then affect the physical condition of a person. In the* Hippocratic Corpus*, two of the four pairs of blood vessels that mainly flow through the internal body cavity end at the nine orifices as well. Another two pairs of blood vessels that mainly flow through the superficial parts of the body end at the feet. At present, it is hard to confirm whether there was a similar theory in Europe at that time to the one that outlines that* Qi* flows through the* Mai*, or whether it is just a coincidence that the routes of the two types of vessels were so similar. However, we were able to come to the realization that the blood vessels described in the* Hippocratic Corpus* were complicated systems involving more than just blood circulation.

## 5. Corresponding Symptoms and Therapies

Besides the routes and trends, the symptoms and therapies corresponding to the dysfunctions of blood vessels or* Mai* were also proposed in the two literatures. In the* Hippocrates Corpus*, the symptom corresponding to problems with the first pair of blood vessels is backache and those corresponding to the second pair are backache and orchialgia. The therapies for symptoms related to these two pairs of blood vessels are bloodletting at both the knees and ankles in this literature. Each* Mai* in the* Cauterization Canon of the Eleven Vessels of the Foot and Forearm* has its corresponding symptoms, which spread all over the body along the cyclic direction of the particular* Mai* and are mostly pain related diseases, totaling to about 80 different types. In spite of the differences between the numbers of symptoms outlined in the two literatures, the incidence of the symptoms corresponding to each* Mai* or blood vessel are related to ailments that occur on each of their specific routes in the body. Both the first and second pairs of blood vessels pass through the back, so backache is the symptom pertaining to their dysfunction. Moreover, the second pair also pass through the testicles, and didymodynia is the symptom pertaining to this pair as well. The* foot Greater Yang vessel *has a similar route to those of the first two pairs of blood vessels; therefore, undoubtedly, the symptoms pertaining to this vessel include backache.

In terms of the treatment techniques for these symptoms proposed by the two literatures, they were very simple compared to those of comprehensive diseases. The* Cauterization Canon of the Eleven Vessels of the Foot and Forearm* only recorded moxibustion as the treatment method without specifying the specific moxibustion position required for each symptom, which showed the special and close relationship between moxibustion and* Mai* [15]. In the* Hippocratic Corpus*, only bloodletting was recorded as the proposed treatment in the four pairs of blood vessels theory. This technique was widely used from antiquity to the late 19th century in Europe. Galen, one of the supporters of Hippocrates, promoted the development of bloodletting as a therapy. He claimed that the balance among the four humors of the body had a bearing on health and that blood was the dominant humor. Bloodletting was required to remove the “excess” blood in order to maintain the balance among the humors and to cure diseases [16]. Although bloodletting was not mentioned in the* Cauterization Canon of the Eleven Vessels of the Foot and Forearm*, we could discover from other literatures that the mindsets and the treatment methods of traditional Chinese and Western medicine were consistent for some time. In China, bloodletting is an old therapy in which a sharp needle is used to prick the skin to cause bleeding, and it is manipulated to promote and balance the flows of blood and* Qi* when the blood stagnates. This practice has a long history, and its origin can be traced back to the Stone Age [17]. In 1963, a polished pyramid-shaped stone needle, was unearthed at the Neolithic site of the Inner Mongolia Autonomous Region, China. It was proved that this stone needle was a tool that was used for bloodletting and cutting abscesses [18]. It was not until the times of* The Yellow Emperor's Classic of Medicine *that the theoretical basis, indications, and contraindications of bloodletting, as well as specific bloodletting positions, tools, and five kinds of bloodletting techniques for treating diseases with different features were all described. In the section that discusses the treatment for backache in the* Suwen *(素问), it is mentioned that the malfunction of the* foot Greater Yang vessel* leads to backache which can be treated by bloodletting at the BL40 point (*Weizhong*, 委中). An article in the* Lingshu *(灵枢) also enunciated that backache, according to different symptoms, was caused by problems of the* foot Greater Yang vessel* or* foot Minor Yin vessel*, which were punctured and bled at the BL40 point as the treatment. This therapy is almost the same as the treatment described for backache due to problems with the first and second pairs of blood vessels. In addition, as mentioned earlier the route of the* foot Greater Yang vessel* is strikingly similar to those of the first two pairs of blood vessels.

## 6. Discussion

As the foundation of early Western medicine, the* Hippocrates Corpus* proposed valuable evidence and all-round guidance for medical theory and practice in the fifth and fourth centuries BC, a period in which Western medicine was still in its infancy. Many treatises, such as* On the Heart*,* On Humours*, and* On Anatomy,* contributed to the later development of Western medicine. They represented the earlier Western medicine's understanding of the human body and the exploration of disease treatment at that time. Medical literatures unearthed from* Mawangdui *and the* Hippocratic Corpus* were compiled by the contemporary physicians. Compared to the documents handed down from ancient China, the contents of these unearthed materials are closer to representing the real thoughts of the ancient physicians, and they reflect the status of Chinese medicine at that time because they have not been processed by later generations. Comparing some of the contents of the* Hippocratic Corpus* with those of the* Cauterization Canon of the Eleven Vessels of the Foot and Forearm*, there are significant similarities between the described running routes and the motives behind the proposed numbers of blood vessels and* Mai*. This indicates that the starting points of Chinese and Western medicine were approximately similar in the early stages of their development, at least in relation to the understanding of blood vessels and* Mai*. Both of these medical traditions stemmed from clinical experience and numerology and could not have just stemmed from the physical existence of human beings based on their anatomies. Under the cover of this seemingly accidental connection lies evidence of the common pace of development of human civilization in the same period across different regions. In Western medicine, the theory of the four pairs of blood vessels was considered to be incorrect and was abandoned by later physicians, following which no further additions or developments were made to it [19]. On the other hand, based on clinical practice, the concept of* Mai* was developed into a more refined system and theory, which were finally completed in* The Yellow Emperor's Classic of Medicine*. The earliest connotation of* Mai *is relatively simplistic. When the theory of* meridians and collaterals* was established, the* Mai* became the* Jingmai *which consist of the 12 tendinomuscular meridians, 12 divergent meridians, 12 principal meridians, and 8 extraordinary vessels. Further, their connections with the* five viscera and six entrails *are valued, and more treatments for corresponding symptoms are recorded.

Besides the theories of blood vessels and* Mai* being comparable, there are many other theories that have similarities between ancient Western medicine and Chinese medicine. The philosopher Empedocles proposed the four elements theory according to which the four basic elements, fire, earth, air, and water, formed all matter in nature. Based on this, the four humors theory was established according to which the body consists of four types of bodily fluids, and health is directly influenced by their balance. The four humors were matched one-to-one with the four basic elements and the four qualities by ancient physicians [20]. This analogic mindset is in accordance with the Chinese* Wu Xing* (五行) system according to which the five basic elements are “mutually overcoming” and “mutually generative” in order to maintain balance. The* Zangfu *(脏腑) networks classify the* Yang* organs (*Zang*, 脏),* Yin *organs (*Fu*, 腑), emotions, senses, and bodily fluids by these five elements. Nevertheless, some Hippocratic writers only accepted the existence of two basic elements, fire and water. They claimed that all animals, including human beings, are composed of two elements that have different functions; these two elements are mutually inhibiting, restraining, and interdependent, and diseases develop when the two elements are in discord. The characteristics of these two elements easily remind us of those of the Chinese* Yin* and* Yang*. Chinese sages suggest that all matter is made of* Yin* and* Yang, *and that these two opposing forces should be kept in harmony to preserve health. In the* Hippocratic Corpus*, a treatise that mainly discussed diet stated that the qualities associated with specific foods could be adjusted by processing the foods. For instance, the warmth associated with meat could be suppressed by marinating it in vinegar. This is in accordance with the processing methods of the Chinese Materia Medica in traditional Chinese medicine.

From the above information, it can be inferred that both Western and Chinese medicine advocated that human beings should be in concordance with nature to help the body achieve homeostasis at the outset. Medicine from both these regions originated from similar philosophical grounds. But in the course of their development, they were embarked on two different paths due to different research methods. Western medicine developed from hypothetical deductions, followed by validation of the hypotheses by experimental researches [21]. In contrast, Chinese medicine put more emphasis on heritage and developed primarily by the inductive method. It gradually neglected the concrete anatomy of the body and regarded harmony within and with the environment and holism as its theoretical foundation. This leads to the conclusion that contemporary Chinese and Western medicine have diverse understandings of the human body, the environment, health, and diseases.

## Figures and Tables

**Table 1:** The comparison between the first pair of blood vessels and the *foot Great Yang vessel*.

Vessels	Running route	Direction
*First pair of blood vessels*	Back of head-neck-either side of backbone-legs-crura-lateral malleolus-feet	Head to feet
*Foot Greater Yang vessel*	Lateral malleolus-crura-popliteal spaces-hip-either side of backbone-back of head-parietal region: (1) forehead-ears; (2) inner canthi-nose	Feet to head

**Table 2:** The comparison between the second pair of blood vessels and the *foot Ceasing Yin vessel.*

Vessels	Running route	Direction
*Second pair of blood vessels*	Head-ears neck-backbone-loins-testicles-thighs-crura-medial ankles-feet	Head to feet
*Foot Ceasing Yin vessel*	Feet-medial ankles-medial crura-medial thighs-testicles	Feet to testicles

**Table 3:** The comparison between the fourth pair of blood vessels and the *forearm Minor Yin vessel*.

Vessels	Running route	Direction
*Fourth pair of blood vessels*	Front head-eyes-collarbones, (1) upper part of upper arms-elbows-palms; (2) lower part of upper arms-armpits-ribs: left-spleen-stomach-penis; right-liver and-stomach-penis	Head to palms and lower abdomen
*Forearm Minor Yin vessel*	Ulnar side of medial forearms-medial cubital fossa-medial upper arms-armpit-ribs	Forearm to ribs

## Supplementary Materials

Supplementary Materials This manuscript is a comparative study based on* Hippocratic Corpus* and* Cauterization Canon of the Eleven Vessels of the Foot and Forearm*. The supplementary materials described the main comparable contents (*Mai* and blood vessels) in these two books, which were discussed in this manuscript.* Supplementary Table 1* described the content about “blood vessels” of* Hippocratic Corpus* in its* On Human Nature*.* Supplementary Table 2* described the content about* Mai* in* Cauterization Canon of the Eleven Vessels of the Foot and Forearm*. The supplementary materials can help readers gain a quick access to the content that we focus on in the two books.
