# Supplementary material for: A Comparative Study on “Mai” and “Blood Vessels” in Early Chinese and Western Medicine: Based on Hippocratic Corpus and Cauterization Canon of the Eleven Vessels of the Foot and Forearm
Source: Evid Based Complement Alternat Med. 2019 Jun 9;2019:7826234. doi: 10.1155/2019/7826234 (PMC6590545; doi:10.1155/2019/7826234)
Supplement: Supplementary Materials — This manuscript is a comparative study based on Hippocratic Corpus and Cauterization Canon of the Eleven Vessels of the Foot and Forearm. The supplementary materials described the main comparable contents (Mai and blood vessels) in these two books, which were discussed in this manuscript. Supplementary Table 1 described the content about “blood vessels” of Hippocratic Corpus in its On Human Nature. Supplementary Table 2 described the content about Mai in Cauterization Canon of the Eleven Vessels of the Foot and Forearm. The supplementary materials can help readers gain a quick access to the content that we focus on in the two books. [file 7826234.f1.docx]

*Supplementary Table 1* ：Content about “blood vessels” of *Hippocratic Corpus* in its *On Human Nature*

*Supplementary Table 2*：The Content about *Mai* in *Cauterization Canon of the Eleven Vessels of the Foot and Forearm*
